# An exploration of parent perceptions of a take-home loose parts play kit intervention during the COVID-19 pandemic

**DOI:** 10.1371/journal.pone.0292720

**Published:** 2023-10-10

**Authors:** Calli Naish, Patricia K. Doyle-Baker, Meghan S. Ingstrup, Gavin R. McCormack

**Affiliations:** 1 Department of Community Health Sciences, Cumming School of Medicine, University of Calgary, Calgary, Canada; 2 Faculty of Arts, Department of Communication, Media and Film, University of Calgary, Calgary, Canada; 3 Faculty of Kinesiology, University of Calgary, Calgary, Canada; 4 School of Planning, Architecture and Landscape, University of Calgary, Calgary, Canada; 5 Alberta Children’s Hospital Research Institute, University of Calgary, Calgary, Canada; 6 Faculty of Sport Sciences, Waseda University, Tokyo, Japan; St John’s University, UNITED STATES

## Abstract

The restrictions introduced in response to the COVID-19 pandemic affected the regular routines of Canadians, including access to play and physical activity opportunities, while limiting social connections. In response to this, a recreation centre created take-home play kits that contained loose parts with the aim of facilitating unstructured play. Between August 2021 and January 2022, ten parents participated in semi-structured interviews via telephone or videoconferencing platforms that captured their experiences of the take-home play kits. Using Thematic Analysis, we identified themes and subthemes reflecting parent perceptions and experiences of the take-home play kit. Three themes emerged: (1 *A forced renaissance of play*; (2) *Bringing unstructured play home*, and; (3) *Parenting is child’s play*. Parents shared how the pandemic resulted in decreased physical activity and social opportunities for their children. The parents described how the take-home play kits supported unstructured play as well as their perspectives on the importance of unstructured play. Parents in our study suggested that a take-home loose parts play kit could be a useful resource to help engage children in unstructured play in both indoor and outdoor environments.

## Introduction

### Benefits of play

Play contributes to the development of emotional resilience and adaptability in response to stress [1], risk taking, problem solving, and autonomy [2], and physical literacy and decision-making [3]. Play, while frequently structured through sports and organized activities, also include child-led activities that are spontaneous and unstructured [4,5]. This unstructured play can support the accumulation of time spent doing physical activity [6,7], improve social, cognitive, emotional wellbeing, and contribute to personal development and learning [8] and academic performance [9]. However, there have been shifts in the nature of children’s play from unstructured to structured activities in response to safety concerns (e.g., such as fear of strangers, traffic, and older children; [10]) The increased use of technology (e.g., TV, computers, laptops, electronic toys, radios, music players, console games, tablets, and smartphones) has also influenced how and where children play [11]. Play environments have evolved as industrialization and urban development have replaced many natural environmental play spaces with built structures such as playgrounds and athletic fields [4]. This limited accessibility to outdoor spaces with changes in lifestyles in urban centres has decreased children’s opportunities for unstructured play [12].

### Loose parts play

The theory of loose parts play supports unstructured play and proposes that the play environment should contain non-traditional play material (e.g., tires, milk crates, and ropes) to facilitate creativity and engagement [13,14]. Loose parts play is the label given to any collection of natural or human-made materials that are flexible in interpretation and that provide opportunities for different types of play to occur [15]. In contrast, toys that have been designed specifically for children’s play may be consumer focused [16], and suggestive of how the item should be played with and by whom [17].

There are several benefits of unstructured loose parts play including increased engagement and duration of physical activity [15,18,19], resiliency [2], and positive developmental opportunities in cognitive, social, and emotional domains [1]. Loose parts play offers increased opportunities for risk-taking, independence, imaginative play while building confidence [2]. Furthermore, it is possible that opportunities to interact with loose parts play could lessen aggressive behaviour, while providing opportunities for creativity and social connection among children [13]. Loose parts play can take place in different environments or contexts to promote child-led unstructured play, and loose parts interventions have been successfully implemented in playground environments [18] and early childcare settings [20,21]. However, there is an absence of literature describing the implementation and effectiveness of loose parts play in the home environment.

### Study purpose

During the COVID-19 pandemic children’s outdoor play [22–24] and opportunities for physical activity [25,26] decreased, while their sedentary behaviour, including screen time, video gaming, and television watching increased [27]. Yet despite the potentially negative impacts these changes pose to children’s health and wellbeing, families have reported increases in children’s unstructured play when access to organized sports and recreational activities are unavailable [28,29]. Additionally, although children’s overall physical activity declined during the pandemic [25,26], increased unstructured play opportunities contributed to children’s accumulation of physical activity despite this lack of access [28,29]. Beyond the accumulation of physical activity, increased opportunities for unstructured play allowed families to reflect on their highly-scheduled pre-pandemic lives, and appreciate flexibility, public spaces, and time to connect with one another.

This study was part of a comprehensive evaluation of a four-year community recreational centre’s program (https://www.vivo.ca) with the aim of increasing physical activity through improved opportunities for “spontaneous outdoor play”. Vivo’s mission was “to get kids outside and moving through unstructured play”, and this mission was realized through several different projects (https://www.vivoplayproject.com). The present study investigated parental experiences of one of these projects–the “Play Kit” program. The “Play Kit” program was designed in response to the COVID-19 pandemic public health measures as a resource for parents to bring unstructured play into their homes when conventional children’s activities were shut down. By helping parents “bring unstructured play home” Vivo hoped to not only provide an opportunity for physical activity, but an opportunity for parents and children to reimagine “play” and experience the many benefits of unstructured play. While other studies have explored the relationships between the use of traditional toys and play at home among children [30], as well as loose parts play in school and child care settings [18,20,31,32], neighbourhoods and other outdoor settings [33] to the best of our knowledge there have been no studies that have investigated a loose parts play intervention based in the home environment.

## Method

### Qualitative approach and research paradigm

Our study utilized a constructivist paradigm, guided by ontological relativism and an epistemologically constructivist lens, to explore parents’ perspectives, experiences, and attitudes towards play. This approach was valuable as it allowed us to acknowledge both that a multiplicity of realities shaped by social and environmental factors exist among parents, and that social experiences inform parent’s individual beliefs, attitudes, and perspectives [34,35]. Additionally, this approach highlights the dialogic nature of qualitative research and emphasizes the value of conversation and shared insights between researcher and research participant [34,35]. We conducted semi-structured interviews to facilitate these types of conversations with parents, which allowed us to ask specific questions pertaining to unstructured play while creating space for parents to share greater detail, depth, and personal sentiment surrounding their interpretations and perspectives of play. We utilized thematic analysis to generate meaningful themes that reflected parents’ shared experiences and perspectives about play and facilitating play during the pandemic.

### Researcher characteristics and reflexivity

Researchers’ identities, including our experiences, beliefs, values, and worldviews informed and shaped the research process. These identities inevitably influenced the interpretive meaning-making process of parent experiences. The research team (CN, MSI, PKD-B, and GRM) comprised of middle-aged adults who identified as white cis-gender men and women, with undergraduate and graduate level educations, from middle-to-high income households, and with experiences as counsellors, child caregivers, coaches, and/or parents. The team included expertise in psychology, sport science, and public health, with all members having a personal and or professional interest in play and physical activity.

### Context

Vivo’s play programs targeted children and adults and included several strategies for promoting physical activity and play [33,36]. Between March 2021 and May 2021, when other in-person programs were halted, Vivo developed take-home “Loose Parts Play Kits” for family use. Four take-home play kit prototypes were piloted to inform the final design of their medium and large sized take-home play kits (S1 and S2 Figs), which included loose parts and materials, some of which were consumable items such as pens, paper, and cardboard (S1 and S2 Tables). The cost of materials was estimated to be $180 CAD for the medium kit and $300 CAD for the large kit. The estimated cost of replacing consumable materials for returned take-home play kits was approximately $8 for the medium kit and $45 for the large kits. The take-home play kits included an inventory of materials and information for parents about facilitating loose parts play, but provided no written instructions about how or where (e.g., indoors or outdoors) these materials could be used.

In June 2021, Vivo was able to launch publicly the take-home play kits which were available to residents of the 17 north central communities located within the recreational centres catchment area. Households could sign out one take-home play kit at time by reserving online with government issued identification and a valid credit card. The take-home play kits had to be collected in-person from Vivo, where parents were able to speak to Vivo staff members, ask questions, review the play kit materials and informational resources (provided in English) and receive guidance about facilitating loose parts play. Initially, families could borrow the take-home play kits for one week, however in August 2021, this was extended to two weeks to allow Vivo staff more time for cleaning and replenishing of the take-home play kit materials. Vivo also implemented a small one-time damage deposit of (0.01 cent) to encourage the return of take-home play kits. They charged parents the full cost of non-returned take-home play kits. During the program, Vivo made minor modifications to the take-home play kit contents based on parent’s feedback. These changes were informed by cost effectiveness and overall practicality of the loose part (i.e., liquid food colouring was changed to colouring tablets to mitigate messes/spills, cardboard tubes were changed to PVC tubes so tubes were more durable, and the weed barrier (cloth material intended for weed prevention) was removed from the kits). Vivo implemented these changes in August and September 2021. From May 2021 to March 2022, 201 (103 large and 98 medium; approximately 18 per month month) take-home play kits were signed out, with 37 households borrowing a play kit on at least one occasion.

### Sampling strategy and participant characteristics

Parents and caregivers (herein termed “parents”) who borrowed at least one take-home play kit and indicated that they were interested in participating in the play kit evaluation when they reserved their play kit online were included in our recruitment plan. A Vivo staff member forwarded the contact information of those interested in the study to a member of the research team, who then contacted individuals via telephone or email. Recruitment began in August 2021 in alignment with Vivo’s capacity to manage participant interest and concluded in January 2022 as the ten interviews provided sufficient depth and detail to develop meaningful and relevant feedback for the evaluation. Between August 2021 and January 2022, seventeen parents from different households indicated they were interested in participating, and ten responded to our team. The sample included nine parents and one grandparent (ages 33 to 65 years), eight of whom were female. Participants identified as Caucasian (5), Middle Eastern (2), Southeast Asian (1) and Ukrainian (1), and all but one participant had two children in their household (ages 20 months to 8 years). Participants received a $25 gift card as a token of appreciation. The University of Calgary Conjoint Health Research Board approved the study (REB: 20–0074). Parents provided informed verbal consent to participate in the study.

### Data collection

A member of the team (CN), trained in qualitative research, conducted semi-structured interviews via telephone or video conferencing. Background literature on play and loose parts play informed the design of the interview guide [13,14,20]. The interview guide contained questions surrounding involvement and experiences with the take-home play kits such as *“Could you describe the family’s usage of the play kit”*; benefits of play: *“What did your child(ren) enjoy most about the play kits”*, and impact of the pandemic on physical activity: *“How was your child’s physical activity impacted at the beginning of the pandemic*.*”* Interviews were audio recorded and ranged in duration of between 45 and 60 minutes.

### Data analysis

Data collection and analysis occurred concurrently [37]. A third-party transcription service (Rev Transcription) transcribed interview audio-recordings verbatim. We assigned pseudonyms to maintain participant anonymity. We provided the option for parents to review their de-identified transcript so they could ensure their responses reflected what they wanted to say. Two parents chose to review their transcript. A team member (CN) with qualitative research experience reviewed and inductively coded transcripts in NVivo version 12 [38] in accordance with the steps for undertaking thematic analysis outlined by Braun and Clarke [39]. These codes were reviewed by a second member of the research team (MSI). CN and MSI reviewed these codes and generated initial themes which were refined by MSI and PKD-B, and then further refined by CN and PKD-B into the final themes. Our rationale for undertaking thematic analysis was to identify recurring patterns found within the interview data aligned with the study aim. Codes generated from the data were organized into predominant and important themes.

### Enhancing trustworthiness

The research team engaged in flexible, analytical, and critical thinking throughout the research process [40,41]. Furthermore, the researcher who conducted interviews facilitated their own reflexive process to identify their personal views and to bring awareness to assumptions in the shaping of the research [40]. Recorded memos and notes were stored in NVivo as an audit trail throughout the data collection and analysis process. The research team (all authors) met weekly during the analysis process and engaged in discussions regarding emerging themes in relation to their world views and lived experiences, particularly within the context of the COVID-19 pandemic, in which we were navigating simultaneously with the parents who participated in this study [40].

## Results

Our analysis identified three themes and 12 corresponding subthemes. The first theme *A forced renaissance of play* considers the experience of parenting during a pandemic, and how parents supported their children’s play and physical activity with limited or non-existent resources. In the second theme *Bringing unstructured play home*, parents described the experience of facilitating unstructured play in their own homes. The third theme *Parenting is child’s play* provides insight into how parents define and conceptualize the benefits of play.

### A forced renaissance of play (theme 1)

This theme highlights how children’s play was impacted by the COVID-19 pandemic, and how parents were forced to adapt their perceptions of play to keep their children engaged and active. Parents spoke to the challenges they experienced following restricted access to public facilities and social gatherings, and to the resources they used, approaches they took, and perspectives they gained in meeting these challenges. These experiences are captured in five subthemes: translational stress and fear, alienating experiences, coping with limited physical activity resources, renewed appreciation of getting outside, parental commitment to play.

#### Translational stress and fear

Parents spoke to how the uncertainty, instability, and unavoidable health advisories of the pandemic created constant stress and fear. They reflected not only on how these feelings impacted their parenting, but how their fear-based parenting impacted their children. One participant, Taylor (female, 41) shared that it was challenging to manage the uncertainty, describing herself and the choices she made for her children as “ridiculously cautious”.

“It was hard because I feel like everything was uncertain and it kept changing all the time. Even borrowing a [play] kit, at the beginning of the pandemic I never would have done that because I was worried about all the touching.”–Taylor (female, 37).

Other participants shared similar sentiments as Tanya (female, 37) explained, “we didn’t let the kids go out for a really long time… we shrank their bubble a lot to make it safe.” Tanya also shared that these fears were unavoidably translated to children,

“You can’t tell a kid to stop doing something for a long period of time, they [become] scared… they would never pick up a leaf [now]. They wouldn’t because they know they’re not supposed to.”

Other parents shared similar sentiments and discussed how these anxieties could be attributed to the news and constant COVID-19 messaging they were exposed to. As Beth explained,

“Because of COVID we are experiencing a huge amount of stress and as parents we are scared… [you need] to separate yourself from the news, from that fear, from that stress, and from the whole universe of things we can’t control.” (female, 37)

Similarly, Anna shared how parental fear impacted her ability to create social opportunities for her children even as children began to return to school and activities.

“I think the messages sent really scared people… When people in a position of power send a message of fear, it does stick… so there are still some lasting fears… people are still really afraid and it’s difficult to arrange play dates. People still don’t want to meet new people and bring, I guess, germs into their circle and it’s really creating sort of a separation. You can only see your friends at school.” (female, 38)

For many parents, COVID-related anxiety persisted even as public health restrictions were lifted, contributing to continued isolation and feelings of guilt.

#### An alienating experience

Parents reflected on how upholding public health measures altered their children’s social interactions, not only limiting their social contacts, but changing how they interacted and who they interacted with. As Taylor shared,

“They were definitely missing those interactions with peers their same age. Even through the summer, we were just being really cautious. It was literally just our family, maybe my sister sometimes, [so] they were having more adult interactions than kid interactions.” (female, 41).

Anna described her son’s disinterest in activities that moved online and lacked the informality of in-person experiences.

“I mean my son was three. They run around in their dance class, and they bump into each other, and they high-five, and they giggle and laugh and just feel each other’s energy.” (female, 38).

Lily noticed similar changes in her grandson’s socializing and described how the decrease in social opportunities affected her grandson’s ability to interact with other children,

“When he is at the playground now, he has to learn how to do that, “play with me” stuff. Playing alongside and then inviting someone to play… He wants to socialize, but he’s not quite sure how.” (female, 65)

Parents also noted a change in their own interactions with their children. Keith (male, 41) described how he had to respond to his children asking, “why can’t we go to [our friend’s] house,” and explain why they were not able to visit with others, especially kids the same age. He emphasized the burden of providing constant COVID-related reminders, explaining that he had to constantly tell them, “wash your hands, wash your hands, wash your hands.” Many parents reflected on their parenting choices and behaviours with a tone of guilt, or wishing they could have made different choices. Lily shared that parents were feeling torn about their COVID-related parenting choices,

“So [parents] look back and think, “Oh gee, have I just cut my kid off from a year’s worth of social growth that I didn’t have to?” or, “was I as safe as I possible could and that’s more important?” I think they struggle with that; they struggle with the choice they made. (female, 65).

#### Coping with limited physical activity resources

Many parents shared how the closure of recreational facilities and the cancellation of organized activities drastically limited their usual physical activity. As Tanya described,

“We’re an "ing" family, I tell people. The "ings." Hiking, biking, swimming… they had dance and gymnastics and [outdoor school] and swimming…we went from having activities every day, basically, to having no activities, and they went from having play dates and playing outside, going to playgrounds and exploring our community, to staying at our house, and we have a beautiful backyard, which we love, but definitely it was a change.” (female, 37)

Some parents struggled with how these restrictions impacted their parenting values, as Anna explained,

“I feel especially [challenged] because we’re trying to start [our kids] off on the right foot, and the first five years of life, we believe, are imperative in setting lifestyle habits. So, to have the pool closed… those things, they have a big impact.” (female, 38).

Parents described how these restrictions forced them to be creative and find unstructured ways to maintain their children’s physical activity,

“[the kids physical activity] hasn’t increased or decreased. I think it’s just kind of shifted. Where before the pandemic it was a lot more organized activities. And then after the pandemic it’s been mostly just free outside. One thing since the pandemic, because everyone’s stuck inside, we just make it a point to go outside every day.”–Andrea (female, 37).

Like Andrea, many parents spoke about their utilization of outdoor spaces to keep their kids active during the pandemic.

#### Renewed appreciation of getting outside

Parents spoke not only to how they utilized outdoor space to engage their children in play and physical activity, but also to how they came to better appreciate these opportunities. Taylor shared how they benefited from an increase in outdoor activity:

“I think one good thing about COVID is we tried to get [the kids] outside more. And because we’re not running around doing the multitude of activities that we used to do, they’re doing more outside activity and more unstructured play outside. As opposed to more structured team sports, now it’s more family bike rides, and it’s running around in the backyard, or going on a hike, or those kinds of things.” (female, 41)

Jess described the challenge of playground closures and an appreciation for the return to “normal” when they were re-opened,

“We live right across the street from a park, so we are at the park every afternoon. So, when the parks were all closed, that definitely affected [us] because we couldn’t use it. I’d take [the kids] on walks and that got old pretty quick… after the parks opened, we were, back to that regular play routine, going back to the parks and using them every day.” (female, 37)

Like Jess, many parents made a point of going to the park or “getting outside” every day, and many parents explained how their families came to value the outdoors more than they had prior to the pandemic.

“Every day and we spent one hour just playing and walking. It’s beautiful. Because we can’t go [places] indoors, we get to benefit from the outdoors, and we get to love the outdoors more than ever. We get to see the benefit and the beauty of nature and appreciate it, right? Because we took for granted the beauty of nature and then when COVID hit and we can’t go indoors, we just appreciate the fresh air, the sky, the sound of birds, the loose parts that nature gives us, right?”–Beth (female, 37)

Some parents shared how even as organized activities resumed, they continued to engage their kids in unstructured outdoor time, rather than register them for their former pre-pandemic activities,

“The weird thing is we haven’t really gone back to organized activities… we haven’t needed it as much as I thought we did. There’s something about that free play that we found valuable over the pandemic. It’s kind of forced us to just play, I guess, rather than do something organized.”–Andrea (female, 37)

While many parents did report that they were excited to be able to send their children back to organized sports and activities, all of the parents acknowledged they found new value in “free play” having gone through the pandemic.

#### Parental commitment to play

Parents explained that in addition to utilizing outdoor spaces during the pandemic they had to utilize whatever resources were available to them to keep their children engaged in play at home. As Beth (female, 37) described it, supporting play requires “providing space and providing time and material. These three things: space, time materials and just encourages them to have fun.” For some parents this required an additional financial commitment as they invested in more toys for their children. Tanya spoke to this commitment and recognized her family’s privilege in being able to make this commitment,

“[we bought] Lego kits. We bought an indoor little trampoline. We have the capacity to buy our way out of some of it, to be honest. We took a two-and-a-half-week vacation to BC both summers. My parents live there so we didn’t have to break the bubble. Having those opportunities was really helpful.” (female, 37)

Other parents spoke to their use of free resources like the take-home play kits, and local adventure playgrounds. Regardless of financial contribution, all parent’s described the extra work that went into supporting their children’s play.

“We do a little obstacle course type thing. Not obstacle courses, but a running, I’m going to time you, run up and down the hallway 15 times. Yesterday, currently we have scarves tied across the hallway upstairs, where he had to jump under and over, that kind of stuff.”–Roberta (female, 41)

“I think this is the way I really made a sacrifice to support their play with mud because it’s huge mess. My husband said, "I feel embarrassed in front of the neighbors, can you stop giving the kids mud to play with. You go play with something nice and neat." But [my son] is a mud monster, and [my daughter] became a mud monster supporter.”–Beth (female, 37)

“For us, it’s taking advantage of what was available. There was so much that wasn’t available. So that was another reason why we grabbed the play kits because there’s not a lot else… it really required a lot of engagement from my husband and I. It required us getting involved in their play and be really hands on. To give them a sense of stability where they feel like free enough to relax and play.”–Andrea (female, 37)

While parents utilized many resources to support their children’s play, their time seems to have been the most fundamental of these resources.

### Bringing unstructured play home (theme 2)

Parents shared their experiences of using the take-home play kit, explaining that it was easy to use, that the parts could be adapted to different environments, and it created an unexpected opportunity for family connection. Importantly they shared that bringing unstructured play into their homes allowed them to reconceptualize play and better understand how to support their children’s play activities. These experiences are captured in four subthemes: versatility supports play, a doorway to independent creativity, appreciating connection through play, assuming the role of “gatekeeper to play”.

#### Versatility supports play

Parents highlighted that they appreciated the versatility offered by the play kits, in both where and how they can be used, especially in the context of the pandemic where space and opportunity were limited. As Beth explained,

“because it has loose parts we can take them outdoors, or we can play indoors,” With the loose parts… the child is getting creative. There’s no wrong or right. He can just explore, he can mix and match. If he’s outdoors, he can just create his own play, right? It’s not adult directed, it’s rather child directed.” (female, 37)

Similarly, Andrea emphasized that because loose parts are not prescriptive, they allow play to be completely changed when they are placed in new environments,

“We played with them outside, we play with them inside, they played with them in the bathtub. They transfer to different environments and then it’s like a whole new way of playing with it all over again.” (female, 37)

Other parents shared that using the play kits their children were able to find new ways to engage in repeat activities,

“The first day they just took everything out and then they were building forts with it, and just decorating the forts with some of the other items in there, that kind of thing. And then the second day was when our friends came over, so they took everything down, and then the same thing with our friends, they built another fort out of it, a different one.” (female, 41)

Parents appreciated that the loose parts provided an open-ended experience. Jess (female, 37) described how her children were excited that she let them “do whatever they wanted” with the play kit. While Lily shared that the play kits were able to entertain her grandchildren in a way that reminded her of how she played when she was a kid,

“They’re not specifically toys, so I like that, that they were just ordinary things that you could use imaginatively as a child. And that’s the kind of things, again, that as a child, I would’ve found that fascinating… I just think that using ordinary things in play is really important because it’s imaginative and it’s not somebody else’s idea of what a toy is. It’s your idea of what a toy is.” (female, 65).

Andrea shared similar sentiments and reflected on her children’s capacity to entertain themselves, appreciating that the play kits left room for this type of independence.

“I could put them in literally any environment, and they would find something to be interested in. It could just be a field of grass and they won’t be bored. They can generate enough play and interest from inside almost that they can just play anywhere with toys, without toys, with things that aren’t meant to be toys. So, I really like just giving them that free play.” (female, 37)

The flexibility of the take-home play kits appeared to be beneficial, supporting play in multiple environments, with little to no parental involvement. Allowing children autonomy as they could take the kit and use it where and how they wished.

#### A doorway to independent creativity

Parents described their children’s excitement when they first brought home the play kits, suggesting that the novelty of the loose parts fostered their children’s sense of exploration. As Lily (female, 65) shared, “they were quite excited, to have this box arrive and see what was in it. Just to have a look at everything that was there.” Tanya (female, 37) similarly explained, “there is like a certain novelty of all this newness, like opening the box and the books,” and further suggested that once the box was opened it becomes “less magical.” Parents noted that although the “newness” may wear off over time, the play kits offered a creative departure from their children’s typically structured lives,

“Their day is so driven by structure and, "Okay, now we have to go here and now we have to go here and do this and do that and do this." I feel like when they’re just given, for example, this Vivo play kit and it’s so open ended, their mind sort of walks through this door and there’s no rules.”–Anna (female, 38)

Parents also shared their appreciation for how the play kits facilitated independent play, as Tanya explained,

“I just appreciate [that] it was all them, it wasn’t me directing it. Right? Or I didn’t really make any suggestions, I opened the box the kids jumped in and started being creative and that kind of thing. I really appreciated that it was accessible to the kids on their own as well, and that they could do it independently, because I think that’s important too. Sometimes kids will ask me, "Well, what should I play?" Or that kind of thing, some kids don’t have as much exposure to play. Right? And how to play, and how to be creative, and how to problem solve.” (female, 41)

Other parents noted that the loose parts allowed children to lead the play even when they needed support from parents. As Beth explained,

“When [play is] child directed it’s more motivating for the child, rather because the adult is not telling him what to do and which direction to go. Here, I provide the material and it’s up to you what to do with them. As long as I’m here to supervise for safety, you are the leader.” (female, 37)

Even for children who were uncomfortable playing on their own, the play kits offered a sense of adult-free exploration. Roberta described how she used the play kit as a tool to help her child play more independently,

“[I look for] anything new and exciting to entertain my boy who’s attached to my hip. My hope was that he might be able to do some of it on his own, or even just for him and I, as a little family unit, just something new and different… [there is] no age limit to the toys in there. For example, it might be a mini little shovel or something that is exciting for a little person to see an adult size or a big kid’s size tool, he’s not restricted to the baby Fisher Price one. It was really cool and exciting; he had never seen a tarp before. So, we put bungee cords and tied a tarp all over the main floor, so it’s just items that we don’t typically either have, or do I give him access to. It’s exciting in that way, different things for him to play with that were real.” (female, 41)

Keith also shared his appreciation for how the novel items in the play kit encouraged children to think creatively.

“I think the biggest benefit is in the box, there’s items that we don’t have. It’s interesting to them because they’ve never seen that before. It’s a bunch of stuff that is not even related. We’ll have kits at home, but it’s all related to each other. But in this, it’s unrelated stuff, completely a surprise thing to them, like a bungee cord or a gardening kit or baking stuff. It’s so many things in one box that they have to now think of what to do with them.” (male, 41).

Many parents noted the value of having these novel experiences at a time when children were growing increasingly bored at home.

“[I wanted] anything that sparks their interest and keeps them engaged through the pandemic shutdown. [The kids] spent a lot of time in their own yard and they have a lot of stuff that they do in there. But it’s kind it nice to have something abnormal show up.”–Lily (female, 65)

#### Appreciating connection through play

Parents shared how the play kits provided an opportunity for parents and children to engage in play together. Some parents noted that collaborative play was not new to their household, but that it was nice to have another avenue for playing with their children.

“My kids get along really well. It’s an important aspect of our family, that they get along and that they are happy together. So, they do play really well with the play kits together, but it’s not necessarily like a new thing or something that they don’t do… [the play kit] is something that brings them together. When we have it as a family, it’s just fun, to be honest with you. Every part of it is fun. It’s cool opening it. It’s cool discovering what’s in it. It’s cool figuring out what to do with it, and I like seeing what they do with it, which is really neat.”–Tanya (female, 37)

Other parents explained that the play kits allowed them to re-engage with their children or find new ways to appreciate their creativity and independence. Andrea explained how she allowed her children use the take-home play kit independently, and then engaged with them after to see how they had played:

“I think when we used the kits outside and there was fort building and cardboard building, that was a chance for both. They needed a little bit of help. So that was a chance for me to help them as well, so there was the connection of doing it together. But also, when I didn’t help them and they kind of built things on their own I was able to appreciate them in a new way for what they can do. And they’d be like, "Mom, look what we built." And it was always awesome. And I think that’s a connection. So, we weren’t necessarily doing it together, but we were able to connect after it was built to like, "Let’s see what you did.", "Wow, this is awesome.", "Cool.", "I like how you did this and that." So that was [a] way for me to connect with them.” (female, 37)

In these ways the play kits facilitated collaborative interactions, and parents were able to both directly and indirectly engage in their children’s play.

#### Assuming the role of “gatekeeper to play”

Parents reflected on the unstructured play experience, how this experience impacted their own perceptions of play, and their own roles in facilitating their children’s play at home. Taylor explained the value of having these types of unstructured play opportunities,

“For my kids it was a new experience, so that was exciting. Again, building that curiosity, and then also that unstructured play, which is super important for kids. So, using their imagination, being creative, and not having something with rules, but just coming up with their own, not necessarily rules, but their own ideas.” (female, 41)

While Luke shared that borrowing the play kit reflected his own interests and the benefits he sees these interests can hold for children as they develop,

“[I] believe it is good to tinker and see that through many, many examples. I’m also an engineer, so I like to tinker myself. I’ve seen how that makes a difference in children, for some of my friends who have older children, all the way when they get to university.” (male, 33)

Parents who had previous experience with loose parts play suggested that the play kit helped to reaffirm the value of unstructured play and unscheduled time,

“I think the kit is just a good reminder of the importance of that unstructured time. And again, just how you can just take those ordinary items and allow the kids to be creative with them and stuff, and just allow them to explore and that kind of thing… a reminder that we don’t need to be busy all the time and just to let [kids] have those moments where they can play with that for a whole afternoon. Instead of all that, we got to do this, and we got to go do this, and we got to plan this, and that kind of thing.”–Taylor (female, 41)

Other parents who were not familiar with unstructured play noted how the play kits changed some of their assumptions about play. Andrea explained,

“[I saw] how things that aren’t toys, just everyday items, can create a ton of creativity and imagination and play. I think just looking in different spots to give [my kids] play and being freer with what’s already in the house. Like, "Okay, you want to take out all of the pots and pans okay, fine." Not to stop them like, "This is not a toy." If it’s not breakable and it’s not going to cause a problem to play with then maybe, it’s okay to play with. It opens up their play because I think it opens up mine. I’m the gatekeeper to play. I’m the one who provides the things. So, it kind of has to impact me first.” (female. 37)

### Parenting is child’s play (theme 3)

In this theme, parents build on their own growth and understanding of what play is or can be by haring their conceptualizations of play, emphasizing the difference between structured and unstructured play. They offered insight into how play is important for children’s social and emotional well-being, as well as their growth and development. Parents spoke to their children’s play experiences both broadly and with the play kits. Parents reflections about play are captured in three subthemes: navigating a definition of play, play needs to be social, play is how children learn.

#### Navigating a definition of play

Parents shared different perceptions of play, some emphasizing the importance of unstructured play, while others identified all play as valuable, even the more structured play activities. Beth described play as not only a learning opportunity, but also as “fundamental” and as a “human right”,

“Play is fun. Play is relaxing. Play is a learning opportunity for kids… Children learn by interacting with the environment, by touching stuff, by getting hands-on. This is a way they will learn. There is no other way. They won’t learn by watching TV, they need to get involved… Engaged in exploring, right? [Play] is everything for a child, it’s [their] job just to play. And I think it’s a human right also. Play is fundamental. I would say it’s fundamental for children’s development.” (female, 37).

Similarly, Lily described play as a “figuring it out” process,

“I think play is go out and play, you go out and figure out something to do. So, I believe strongly in free play that they go out and imagine things.” (female, 65).

Other parents noted that structured play, and goal-oriented play was also valuable to children. As Roberta explained that play is inherently fun, regardless of the amount of structure involved, and emphasized the importance of creating balance in providing both types of play,

“You [can] use your imagination in structured fun. If you use your imagination, no structure or structure, that’s still fun. Like if you’re building a puzzle, there’s a structure to it in that you have a task in mind, or a goal in mind, but it’s still fun to do it. Play is fun whether structured or unstructured…. I think both [structured and unstructured play] have benefits, I know I’m too goal oriented, structured. I really have to make a point of [saying], “it’s okay to be unstructured,” because I don’t want to teach my kid only this way. I really try hard to make it unstructured when we can.” (female, 41).

Tanya also reflected on the relationship between structured and “authentic” play,

“I don’t know if I know exactly what "play" is. I think it’s authentic and un-guided and joyful, and engrossing. Like when my kids are really playing, they are engrossed in whatever they’re building, whatever they’re creating, whatever they’re imagining. Usually, it’s something that they have really thought of or taken or been inspired by. My kids love building the [Lego] sets. Like they get the book, it tells them how to build the Disney Princess Lego set, and they do that. It is a form of play, but it’s very guided… It’s not just like "here’s a bunch of Lego blocks and build whatever you want." I consider that authentic play…” (female, 37)

Parents also spoke to learning about play themselves and coming to new conclusions about the meaning of play. When providing a meaning of play Andrea shared,

“I’ve heard some things about play, so this is things that I’ve learned and it’s probably not conclusions that would come to on my own, but I have come to understand play as activities that are not outcome based.” (female 37).

Parents’ descriptions of play highlight play as imaginative, child-led, and unrestrained, but also as an opportunity for experiential learning and developmental.

#### Play needs to be social

When discussing the benefits of play, many parents described their children’s experiences during the pandemic, speaking to the lack of interaction they had with other children. They emphasized that playing with others was important for developing communication skills, and for their children’s overall daily enjoyment and well-being. Roberta explained how her son’s cautious nature often limited his playing, how his friends helped him engage in play activities, and he struggled when he couldn’t interact with his peers,

“He’s a very cautious kid, and doesn’t like to do things, but with his friends, he’s a beautiful maniac and plays well with them, everything is really fun. Right now, he’s supposed to be at preschool, but he’s not there. He can’t play with friends… Yesterday I was able to take him to a field, an empty field with no one around, threw this airplane around to chase it and that was good, but that’s not six hours, right? It’s a challenging week for sure”. (female, 41)

Lily shared similar challenges regarding her grandson and his ability to play with others, and how this socialization could not be replaced by parents at home, regardless of the resources they utilize to keep their children engaged in play.

“He wants to socialize, but he’s not quite sure how. I think that’s the biggest impact I would say. Again, I think my daughter and her husband have done a great job of trying to keep them engaged and they have a climbing frame, and they have all this stuff in their yard and dug holes and make mud pies and do all that kind of stuff. But it still is [the kids] all the time with their parents.” (female, 65)

Other parents reflected on play and socialization more broadly, suggesting that play inherently involves enjoyment in interacting with others. Tanya explained how she understands the benefits of play,

“I think for me play is about imagination and creativity and interacting with other people. I just feel like it’s just about that interaction with others mostly, I guess too using your imagination. And I think it has a lot to do with having fun and the enjoyment you get out of this.” (female, 37)

Tanya also suggested that often we ignore that play is more than just an opportunity for imagination and creativity,

“I think it definitely is an imagination, I think that’s where imagination and creativity come from… Definitely creativity, but also, I think play is more. I don’t think we count how therapeutic it is for kids.” (female, 37)

Parents discussion of socializing through play suggests an understanding among parents that play “belong” in a sense to children, and that they’re playing together is a space for not only social development and growth, but also for emotional comfort and social support.

#### Play is how children learn

Parents described play as an imaginative process that helped children to learn and develop problem solving skills through creativity and exploration. As Jess explains,

“[Play] lets them kind of test their own boundaries. Right? They can figure things out. They can work on their imagination.” (female, 37)

Roberta also discussed the importance of imagination, suggesting that imaginative play helps children to develop resiliency,

“[Play is] imaginative, using different parts of your brain, different synapses. I think it’s important not to be so high strung that something has to be completed or there’s a goal, or there’s a structure. I think it’s important for brains to be able to access imagination, you need ideas to create things. That stuff is important down the road.” (female, 41)

Andrea offered similar sentiments, sharing that she hoped her children would carry their adaptability and creativity into adulthood,

“They can generate enough play and interest from inside almost that they can just play anywhere with toys, without toys, with things that aren’t meant to be toys. So, I really like that about just giving them that free play. … And I’m guessing as they get older, I’m hoping, that that will continue into their adulthood. That they’re not waiting almost for someone to tell them what to do and how to do it. That they’ll have enough creative energy.” (female, 37)

Keith suggested that access to different tools and toys, such as those offered in the play kits, allow children to generate ideas, and that this is an essential element of play,

“Play means, I think, for them is to look at a different tools and look at different toys that they can take out of that box and come up with ideas of how to utilize them and make things out of them or come up with ideas.” (male, 41).

Beth also emphasized the importance of children learning through the hands-on experiences of play, likening a child playing to someone investigating in a lab,

“I think if a child is deprived from play, it won’t benefit his development in a long term, right? So, they develop throughout a lifespan and play is fundamental because they get involved, hands-on, their sensory, their imagination, their cognitive abilities, everything, it’s huge. Like it’s like someone in a lab, right? [Their] totally engaged, totally focused.” (female, 37)

Parent’s descriptions of play, their beliefs surrounding the importance of play, and their reflections on how play was impacted during the pandemic, reflect a new depth of understanding about what play means to a child’s life, and how opportunities for play are invaluable not only to their development, but to their overall well-being.

## Discussion

The aim of our study was to investigate parental experiences of a take-home loose parts play kit during the COVID-19 pandemic public health restrictions. Three themes emerged from the interviews we conducted with parents: *A forced renaissance of play*, *Bringing unstructured play home and Parenting is child’s play*. The COVID-19 pandemic disrupted family routines, impacting work, school, and extracurricular activities as parents navigated the changing public health pandemic restrictions. These changes were often a source of anxiety for parents, and many felt this anxiety was translated to their children. In acknowledging these shifts, parents recognized that their children needed access to play opportunities, and reflected on how challenging it was to provide these play opportunities during the pandemic. Families experienced limited social interactions with others, and decreased opportunities for physical activity; however, parents spoke to how the COVID-19 public health restrictions had shifted some of their perspectives on the busy, activity-filled lives they were previously leading. Parents shared that there were unexpected benefits to the pandemic as they established new relationships with unstructured play, especially in outdoor spaces. They reflected not only on the value of play for their children’s well-being, but also on the challenge of creating opportunities for play, noting the increased responsibility they were burdened with when usual resources were unavailable. The take-home loose parts play kits alleviated some of this burden by providing a novel means of bringing unstructured play home and allowing both children and parents to re-engage with play in new ways. These findings align with previous qualitative evidence suggesting children engaged in unstructured play in response to the pandemic [28].

Loose parts play, facilitated by the take-home play kits, provided children with opportunities to develop autonomy [2], social connection [1], and problem solving skills [2], all experiences highlighted by others studying the benefits of unstructured play. Parents highlighted that the versatility of the play kits was essential to their value, and commented on the versatility of loose parts play in general, suggesting that this type of unstructured play inspired creativity and exploration. Many parents noted that embracing a lack of structure in play was helped in part by the pandemic when they were forced to reframe some of their understandings of play. Parents acknowledged their role as “gatekeepers to play”. Some developed a definition of play, while others refined their definition of as a space for their children to build, create, engage in critical thinking and foster social connectivity in an environment that is unguided, joyful, and facilitated through the use of ordinary items in an unstructured environment. Interestingly, despite differences in their family structures, children’s ages, or pre-pandemic activities the parents’ descriptions of play were remarkably similar and notably, the parents’ understanding of play aligned with existing definitions of play offered by scholars [4,5]. Parents highlighted the value of play for their children and shared how they accessed resources such as the take-home play kit in an attempt to continue to facilitate play opportunities for their children during the pandemic. However, they also commented on the aspects of unstructured play that they were holding on to as public health restrictions were lifted, and life began to return to “normal”. Some parents shared that they would be returning to fewer sports and activities to maintain some of the “slowed down” lifestyle they had established during the pandemic, and others suggested they may not return to organized sports at all, having found their children didn’t need as many structured activities.

Congruent with previous Canadian findings [42], parents in our study reported increased stress and fear, and barriers to finding physical activity options for their children due to the restrictions placed on organized sports and recreation facilities during the pandemic. Not surprisingly, the pandemic resulted in fewer opportunities for both structured and in some situations unstructured physical activity when parks were closed [43]. Nevertheless, parents in our study discussed how they adapted to facilitate play opportunities for their children including spending more time playing with their children, purchasing new toys, and trying to utilize external resources such as playgrounds, libraries, and the take-home play kits. Parents also reported how they undertook more outdoor activity, citing a renewed appreciation for outdoor spaces for both mental and physical well-being. Our finding corroborates the findings of Moore et al.[26], where parental participation was associated with more indoor and outdoor physical activity, outdoor play, and family physical activity during the pandemic. While parents took on larger roles in their children’s play, the implementation of pandemic-related restrictions including limited or banned gatherings, school moving online, cancelled sports seasons, and closure to public recreations centres and parks limited opportunities for children to gather and play together. The pandemic inhibited peer interactions [25,28], yet other opportunities for social connection emerged during the pandemic [24]. Parents in our study discussed how children were unable to connect with their friends and peers, encouraging them to be more intentional about spending time engaging with their children. This led to parents seeking out play opportunities in their communities, such as obtaining and using the take-home play kits. The take-home play kits, in particular encouraged family social interactions as both children and their parents navigated play using the loose parts.

The goal of take-home play kits was to facilitate unstructured play using loose parts, and to do so at a time when parents had limited resources and means to keep their children engaged in play. Loose parts play initiatives, implemented in schools and childcare settings, have shown promising results in supporting physical well-being [18,19,32], socialization and resilience, creativity, risk taking, problem solving, autonomy, and leadership [2]. To our knowledge, this is the first investigation of a recreational facility implemented home-based loose parts play intervention. Our findings suggest that this intervention had a positive effect on not only children’s play at home, but on parental perceptions of children’s play, and the value of unstructured time. Parents reported that the take-home play kits helped them facilitate unstructured play, created an opportunity for connection and collaboration, and challenged existing conceptualizations of play objects. Further, the take-home play kits facilitated unstructured play both indoors and outdoors, helping parents to utilize outdoor spaces and challenge their perception of which spaces are “play spaces”. Our findings are promising given that unstructured play can promote physical, psychological, and social benefits [6–8]. Interventions that support loose parts play, such as using the play kits at home, may also be an alternative option to unstructured play when there are paternal concerns of child safety [10] or when accessibility to play spaces is limited [12]. This take-home play kit intervention might also be a feasible intervention for encouraging unstructured play among children during future pandemics that restrict normal daily activity. Moreover, parents’ reflections about play and unstructured time suggest that opportunities for unstructured play may be appreciated by families even outside of the COVID context. Considering that Vivo delivered the take-home play kits free-of-cost to the community, and they were successfully received by community members, this type of intervention may offer a means of providing accessible unstructured play opportunities for families who may otherwise struggle to access resources. Additionally, parents reflections about the benefits of unstructured play and unstructured time during the COVID-19 pandemic may suggest that increased accessible unstructured play opportunities may be of greater benefit now that they were pre-pandemic.

## Limitations

Our findings were derived from in-depth descriptions of experiences and perspectives of parents using the take-home loose part play kit during the COVID-19 pandemic. Our aim and interview questions were grounded in previous knowledge and evidence, our participant recruitment, interviews, analysis, and interpretation of findings involved reflexivity and included strategies for ensuring trustworthiness [40,41]. While using a qualitative approach does not allow our findings to generalize to a broader population, it allowed us to gain insight into the shared experiences of a small group of individuals living within a specific context. Our findings might inform the design and implementation of similar home-based loose parts play interventions. Moreover, our finding do not capture play as experienced by the children themselves but rather captured the experiences of children’s play as perceived by their parents. While our findings suggest that the take-home play kits supported unstructured play, it is possible that parents were motivated to seek out play opportunities for their children, and thus would have facilitated play activities even in the absence of the take-home play kit intervention. Thus, there is a need for more research into loose parts take-home play kits, including qualitative studies investigating child experiences and quantitative studies (e.g., randomized controlled trials) estimating their effect on increasing unstructured and overall levels of play.

## Conclusion

Our study investigated parental experiences and perspectives regarding a novel take-home loose parts play kit intervention on children’s play during the COVID-19 pandemic. The pandemic negatively affected regular play and physical activity routines, yet parents identified and created opportunities for their children to accumulate play and subsequently physical activity, by using the play kits. The take-home play kits provided opportunities for unstructured play and social connection. Given the environmental and societal constraints that often inhibit children’s play, home-based loose parts play kits have the potential to support and encourage unstructured play.

## Supporting information

S1 Fig Large play kit. (TIF)

S2 Fig Medium play kit. (TIF)

S1 Table Large play kit inventory list provided by parents. (TIF)

S2 Table Medium play kit inventory list provided by parents. (TIF)
