# Supplementary figures and images for: An exploration of parent perceptions of a take-home loose parts play kit intervention during the COVID-19 pandemic
Source: PLoS One. 2023 Oct 10;18(10):e0292720. doi: 10.1371/journal.pone.0292720 (PMC10564120; doi:10.1371/journal.pone.0292720)

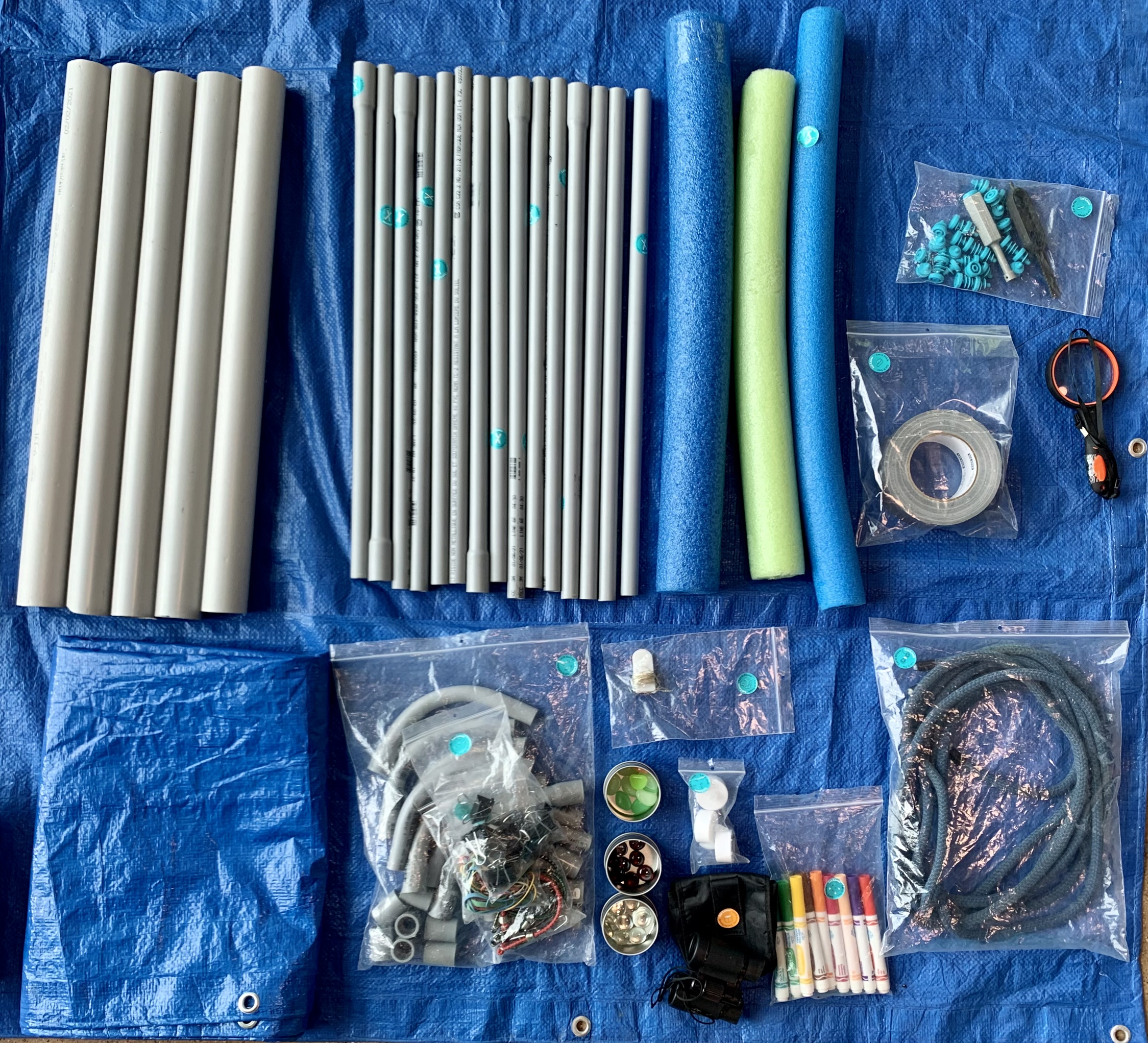

Supplement: S1 Fig — (TIF) [file pone.0292720.s001.tif]

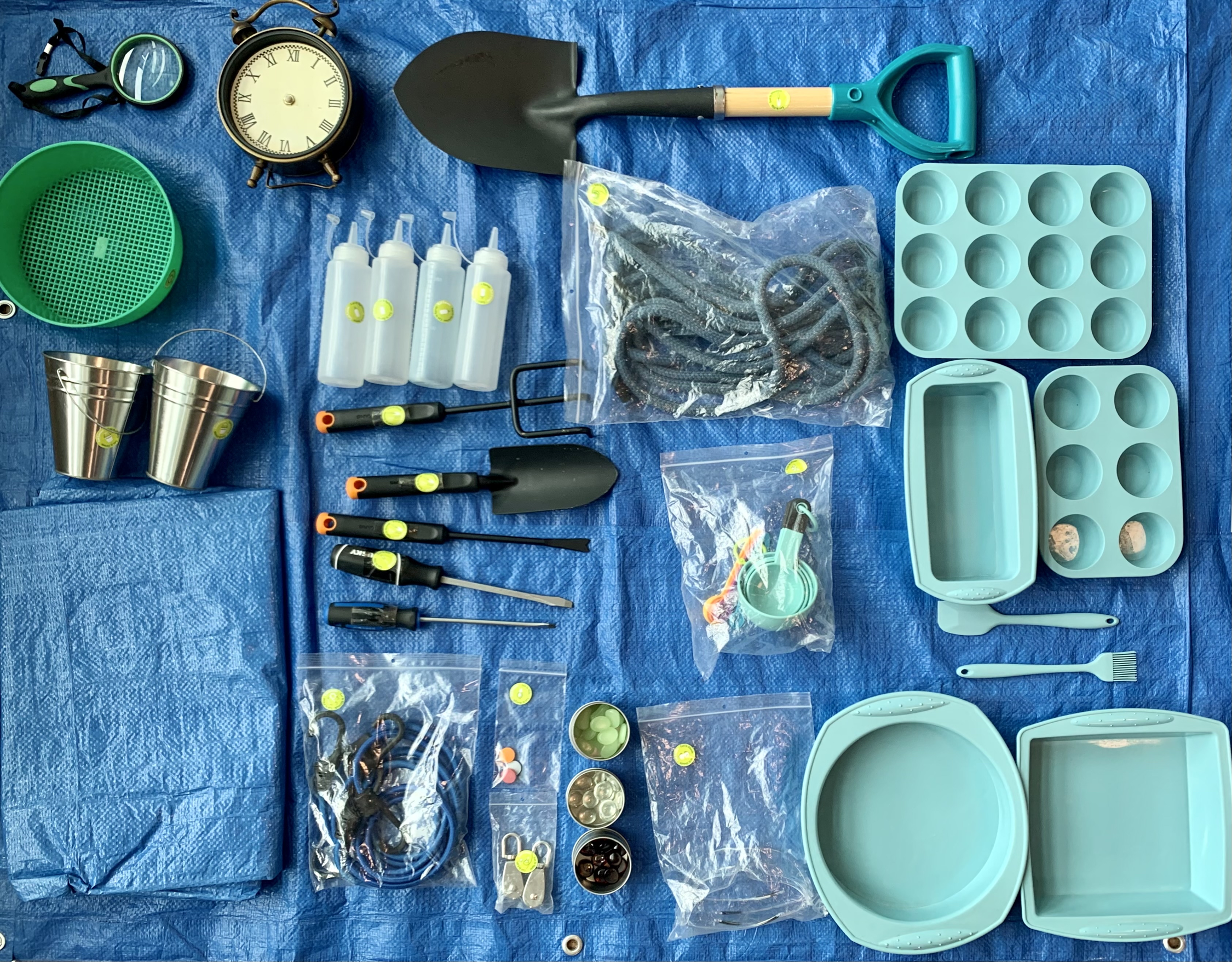

Supplement: S2 Fig — (TIF) [file pone.0292720.s002.tif]

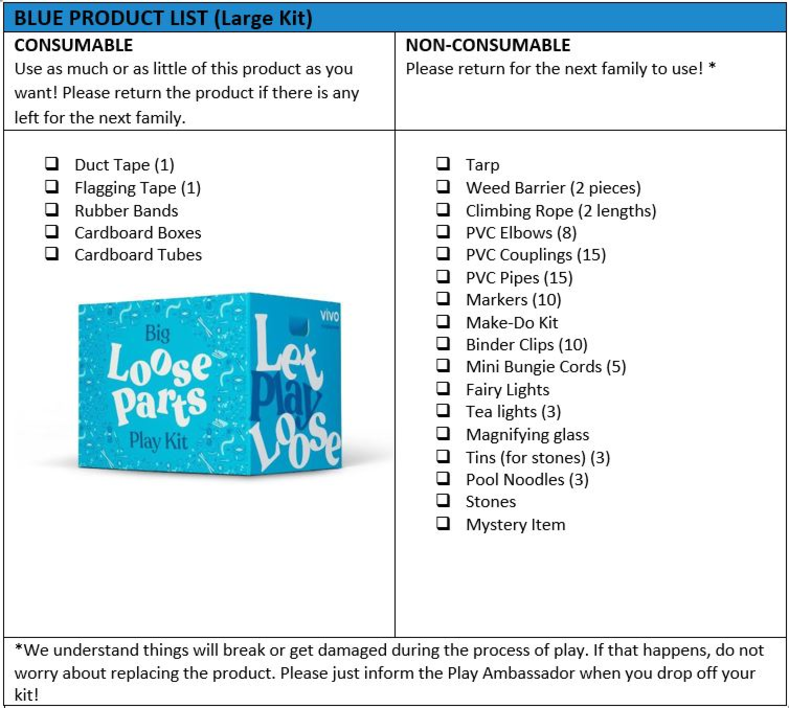

Supplement: S1 Table — (TIF) [file pone.0292720.s003.tif]

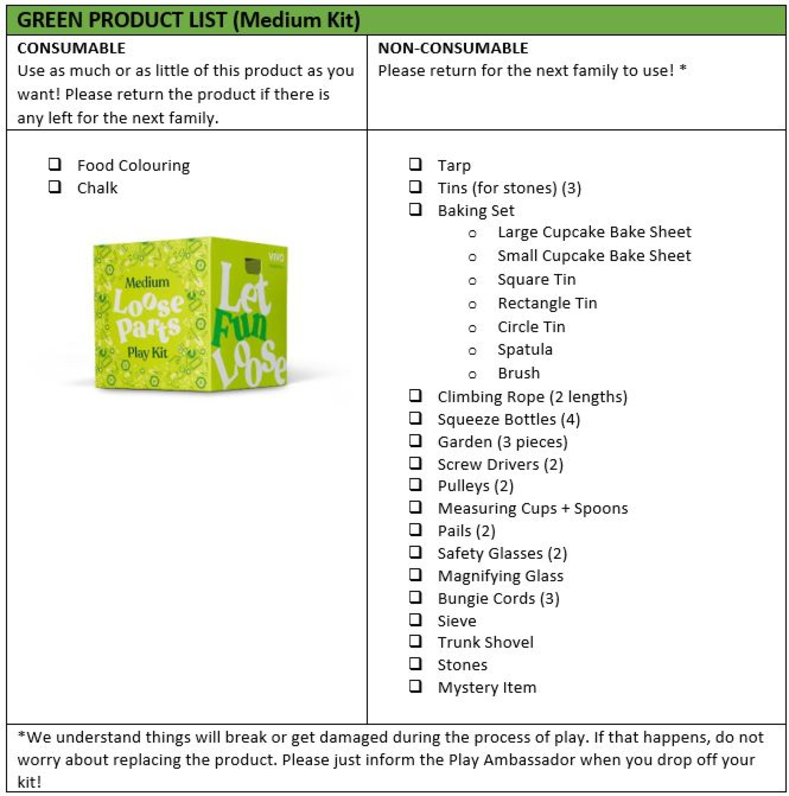

Supplement: S2 Table — (TIF) [file pone.0292720.s004.tif]
